# Interaction of Methylene Blue with Severe Acute Respiratory Syndrome Coronavirus 2 Envelope Revealed by Molecular Modeling

**DOI:** 10.3390/ijms242115909

**Published:** 2023-11-02

**Authors:** Ilya Kovalenko, Ekaterina Kholina, Vladimir Fedorov, Sergei Khruschev, Ekaterina Vasyuchenko, Gennady Meerovich, Marina Strakhovskaya

**Affiliations:** 1 Faculty of Biology, Lomonosov Moscow State University, Moscow 119234, Russia; ikovalenko78@gmail.com (I.K.); tenarra1@gmail.com (E.K.); xbgth@yandex.ru (V.F.); styx@biophys.msu.ru (S.K.); vasyuchenko.katya@gmail.com (E.V.); 2 Scientific and Educational Mathematical Center «Sofia Kovalevskaya Northwestern Center for Mathematical Research», Pskov State University, Pskov 180000, Russia; 3 Prokhorov General Physics Institute of the Russian Academy of Sciences, Moscow 119991, Russia; 4 Institute for Physics and Engineering in Biomedicine, National Research Nuclear University “MEPHI”, Moscow 115409, Russia

**Keywords:** methylene blue, SARS-CoV-2 envelope, viral membrane, structural proteins, electrostatic interactions, Brownian dynamics, coarse grain modeling

## Abstract

Methylene blue has multiple antiviral properties against Severe Acute Respiratory Syndrome-related Coronavirus 2 (SARS-CoV-2). The ability of methylene blue to inhibit different stages of the virus life cycle, both in light-independent and photodynamic processes, is used in clinical practice. At the same time, the molecular aspects of the interactions of methylene blue with molecular components of coronaviruses are not fully understood. Here, we use Brownian dynamics to identify methylene blue binding sites on the SARS-CoV-2 envelope. The local lipid and protein composition of the coronavirus envelope plays a crucial role in the binding of this cationic dye. Viral structures targeted by methylene blue include the S and E proteins and negatively charged lipids. We compare the obtained results with known experimental data on the antiviral effects of methylene blue to elucidate the molecular basis of its activity against coronaviruses.

## 1. Introduction

The basic dye methylene blue (MB) is a synthetic phenothiazine derivative. In the positively charged oxidized form, MB exists as an organic chloride salt, 3,7-bis(dimethylamino)phenothiazin-5-ium chloride. The visible absorption spectrum of MB in water shows two absorption bands with maxima at 615 and 665 nm [1]. Good solubility in water at room temperature, together with partial lipid solubility, planar structure, relatively low molecular weight, positive charge, and spectral characteristics, determine the biocompatibility, properties, and diverse applications of MB in biology and medicine [2,3,4].

MB molecules, which carry a single formal positive charge, bind to the negatively charged biological moieties. They stain nucleic acids [5], cardiolipin-containing membranes [6], bacterial cell walls [7], and viral plaques [8]. In cells, MB co-localizes with the nucleus [9], followed by mitochondria and lysosomes [9,10,11]. 

In clinical therapy, MB was the first antiseptic ever used among synthetic compounds and dyes due to its outstanding antimicrobial properties [3]. MB exhibits antimicrobial activity against a wide range of pathogens, both non-photodynamically without photoactivation [3,12,13,14,15] and when excited at the appropriate wavelengths in photodynamic reactions [12,16,17,18,19,20]. Photoexcited MB produces singlet oxygen with a quantum yield of about 0.5 [21]. The mechanism of photodynamic virus inactivation is highly dependent on the virus type. Nevertheless, viral nucleic acid is considered to be the major target for MB-induced antiviral action [22]. The attack of singlet oxygen induces damage to nucleic acids which can occur via the oxidation of guanosine, the formation of nucleic acid–protein cross-links, and strand breakage [23,24]. In enveloped viruses, proteins and unsaturated fatty acids of the virion envelope are highly sensitive to photooxidation. The main types of photodynamic damage to viral envelopes include the formation of protein cross-links; the complete loss of proteins; and changes in protein conformation, molecular weight, and charge [25]. As a result of such damage, the virus loses its ability to replicate, to bind to host cell receptors, and to fuse; i.e., almost all key stages of the viral life cycle can be disrupted. The photodynamic potential of MB in combination with visible light is successfully used in plasma decontamination with the THERAFLEX MB-Plasma (Macopharma) system [26]. Photodynamic therapy with MB shows positive effects in the treatment of plane warts [27] and herpes lesions [28,29].

The dual antiviral activity of MB can be clearly demonstrated in the inactivation of coronaviruses. In the light-independent manner, MB at very low micromolar concentrations is able to inhibit the replication of several coronaviruses in host cell cultures [12,15,30]. For 10^2^ TCID_50_/mL of SARS-CoV-2, the MB inhibitory concentration IC_50_ was found to be about 0.7 µM [30]. Interestingly, MB is effective in both entry and post-entry replication phases of SARS-CoV-2 infection in Vero E6 cells [31]. According to our data, the short 10 min incubation with MB at concentrations of 1–5 µM reduced bovine coronavirus (BCoV) titers by three orders of magnitude from the initial titer of 10^5^ TCID_50_/mL, leading to a decrease in the virus size, structure distortion, and poor spike resolution in electron micrographs. The incubation of BCoV with MB and further light-emitting diode (LED) 660–665 nm irradiation (photodynamic treatment) resulted in the further reduction in BCoV titers up to a total loss of virus infectivity and increasing damage to the viral envelope [12].

It is clear that the diverse activity of MB against microbial pathogens is determined by several different mechanisms of binding to external structures, penetration through envelopes and cell walls, and reaching targets and facilitating their inactivation. The positive-sense single-stranded RNA genome of SARS-CoV-2, packaged by the nucleocapsid (N) proteins, is protected by a lipid envelope [32]. Recently, we created an electrostatic map of the external surface of SARS-CoV-2 and found a highly heterogeneous distribution of the electrostatic potential field along the viral envelope [33]. In the SARS-CoV-2 envelope, negatively charged lipid domains in the viral membrane and negatively charged areas at the junction of the spike (S) protein stalk and head are responsible for the pronounced patches with negative electrostatic potential that can attract cationic dyes. Also of interest is the ring of negative electrostatic potential around the entrance to the pentameric channel of the envelope (E) protein, facing the outer surface of the viral envelope, formed by negatively charged glutamate residues and surrounding lipids. The most abundant membrane (M) proteins carry an overall positive charge and tend to colocalize with negatively charged lipids. In our model experiments [33,34], we found several MB binding sites in different S protein domains involved in the initial processes of the viral replication cycle. Thus, MB was able to penetrate into the pocket of the S protein head formed due to the transition of the receptor binding domain (RBD) into the “open” state. We supposed that the binding of MB to the RBD of the S protein may prevent the interaction of the S proteins with the receptors on the host cell [35], thereby reducing viral infectivity.

In this study, we aimed to find the sites of MB action on the SARS-CoV-2 envelope which consists of protein and lipid components. Standard docking methods are hardly applicable as they assume that the approximate position of the binding site is already known, and it is only necessary to determine the position of the ligand within this site. A global search for ligand binding sites using standard docking algorithms over such a large surface, which is the coronavirus envelope, is now computationally infeasible. To reduce the computational complexity of such a problem, we use the Brownian dynamics (BD) method to find exactly those energetically favorable configurations of molecules that can be easily reached in the process of Brownian diffusion. The BD method provides high computational performance due to the use of an implicit solvent model, rigid bodies for all participating molecules, and simplified electrostatics. The approximations used in the BD model make it possible to reproduce the processes of the diffusion and electrostatic interaction of molecules, leading to the formation of a collision complex. The use of coarse-grained (CG) rather than all-atom models in BD leads to an extra increase in calculation performance without impairing the accuracy of the results obtained. Further transformation of the encounter complex can be studied using all-atom models and remains beyond the scope of this study.

Recently, we used this approach to identify binding sites for photosensitizer zinc phthalocyanine with the coronavirus envelope and demonstrated its effectiveness [34]. Here, we apply this approach to study the interaction of MB with the SARS-CoV-2 envelope, identify MB binding sites on the SARS-CoV-2 envelope, and discuss the simulation data in relation to the known MB antiviral effects and possible underlying mechanisms.

## 2. Results

**Figure 1:**
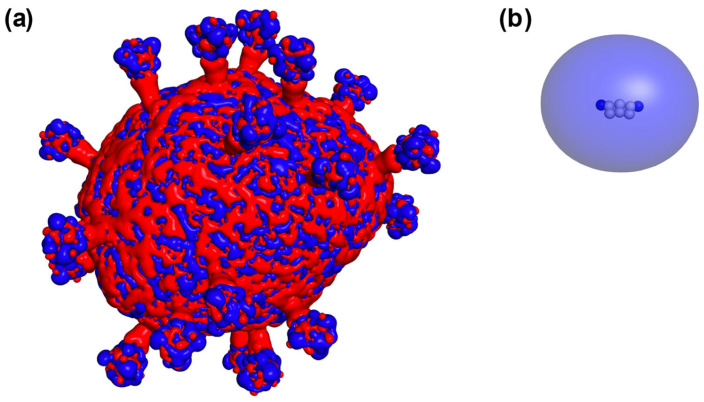
Electrostatic potential surfaces of the SARS-CoV-2 envelope (**a**) and MB (**b**) colored by red (–1 mV) and blue (+1 mV).

The electrostatic potential field of the SARS-CoV-2 virus envelope shows remarkable heterogeneity, with prominent regions of both positive and negative potential (Figure 1a). These negative regions of electrostatic potential primarily originate from negatively charged lipids and specific negatively charged amino acids within the S and E proteins, whereas positive patches are generated by the M proteins. MB has positive electrostatic potential (Figure 1b).

**Figure 2:**
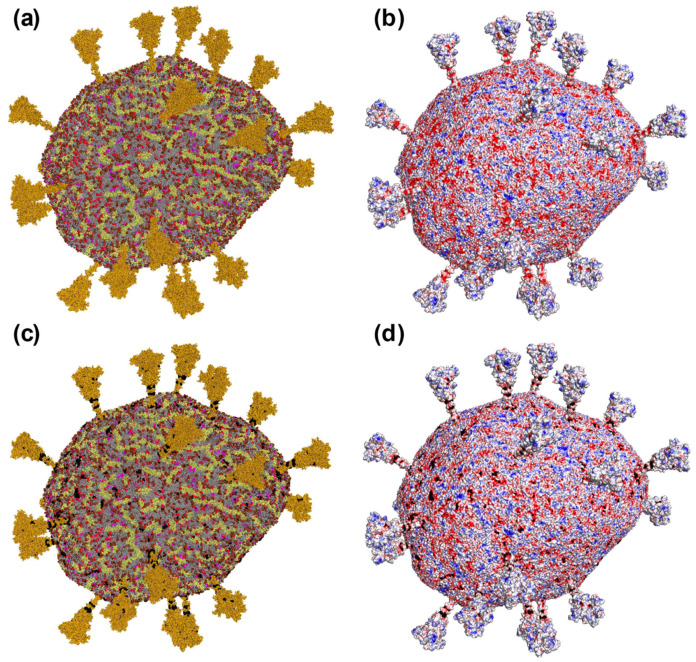
Binding of MB to SARS-CoV-2 envelope. (**a**) Surface of the virion envelope model, colored in accordance with the envelope component types: neutral lipids are shown in gray; negatively charged lipids are colored in different shades of red (POPI molecules—in brown; POPS—in magenta; CDL2—in red); predominant proteins are colored in different shades of yellow (the S proteins—in ochre, the M proteins—pale yellow); shown copy of the E protein is colored in white. (**b**) Surface of the virion colored by its electrostatic potential from −50 mV (red) to +50 mV (blue). (**c**,**d**) Electrostatic encounter complexes of MB and the viral envelope with the energy of electrostatic attraction of more than 2 kT. In (**c**), the color of the components of the virus envelope is the same as in (**a**). Centers of mass of MB are visualized as small black spheres.

**Figure 3:**
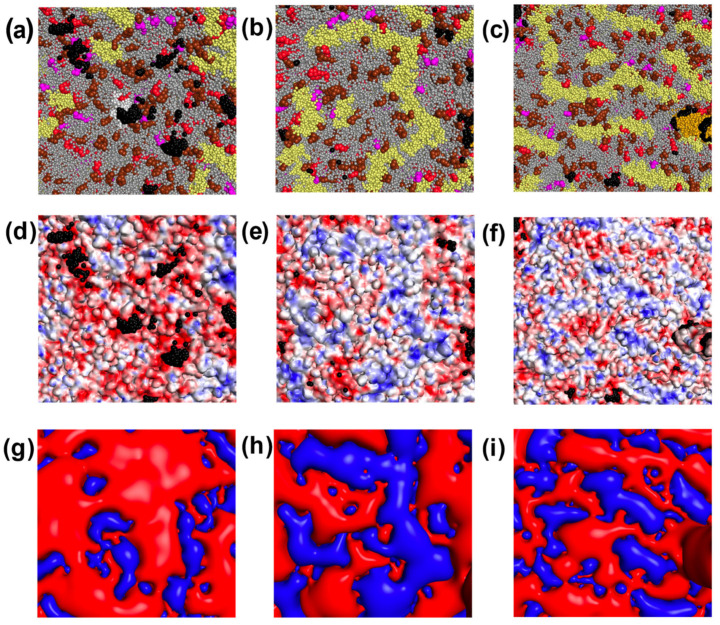
Fragments of the SARS-CoV-2 envelope with different MB binding abilities: fragment 1 (**a**,**d**,**g**) with high MB binding ability; fragment 2 (**b**,**e**,**h**) and fragment 3 (**c**,**f**,**i**) with low MB binding ability. In (**a**–**c**), the components of the virus envelope are colored the same as in Figure 2a. In (**d**–**f**), the surface of the virus envelope is colored by its electrostatic potential from −50 mV (red) to +50 mV (blue). Centers of mass of MB molecules are visualized as black spheres. In (**g**–**i**), the equipotential electrostatic surface –1 mV generated by electric charges of the envelope is colored in red, and the equipotential electrostatic surface +1 mV—in blue.

We analyzed 33 thousand electrostatic complexes of MB with the SARS-CoV-2 envelope with the energy of electrostatic attraction of more than 2 kT, obtained as a result of independent computational BD experiments (Figure 2c,d). The MB model carries two partial positive charges localized on the methylamine groups. As one can assume, the binding of MB occurs through the electrostatic interaction of these groups with the negative charges of the protein and lipid components of the viral envelope. It can be seen that the binding sites of MB on the envelope are spotty (Figure 2); in particular, there are both areas with high binding ability (Figure 3a) and vast areas on the envelope where MB does not bind at all (Figure 3b,c).

Figure 3a shows that the E protein, visualized in white, is an attractive target for MB binding. Glutamates of the E protein pentamer exposed to the viral membrane surface create a prominent area of negative potential (Figure 3g). In addition, the E proteins tend to co-localize with negatively charged lipids, enhancing the local negative electrostatic potential around the E proteins. Figure 3a shows clusters of negatively charged lipids consisting of cardiolipin (CDL2), palmitoyl oleoyl phosphatidylserine (POPS), or palmitoyl oleoyl phosphatidylinositol (POPI) molecules. Compared to separately located negatively charged lipids, the clusters form significantly more intense negatively charged regions that effectively attract MB molecules. Note that the positively charged M proteins are relatively rare in such regions.

The existence of vast areas without the ability to bind MB (Figure 3b,c,e,f) seems to be surprising, since the coronavirus envelope contains a large number of negatively charged lipids almost evenly distributed throughout the membrane, which, in principle, can effectively attract a positively charged MB. However, as can be seen from the distribution of electrostatic potential on the surface of the SARS-CoV-2 envelope (Figure 3e,f), in these regions, there is a significant number of positively charged patches generated by the M proteins (shown in pale yellow in Figure 3b,c) which prevent MB molecules from binding negatively charged lipids adjacent to them since the interaction of MB with such membrane fragments cannot achieve electrostatic attraction energy of 2 kT or more. These patches could be clearly identified by vast blue equipotential surfaces in Figure 3h,i.

We calculated the number of MB interaction events with each component of the coronavirus envelope (Table 1). In the case of simultaneous contact of a MB molecule with several coronavirus envelope molecules, we counted this event for each type of envelope component involved. As a result, the sum of the event ratios exceeded 100%. Overall, MB interacted with coronavirus envelope proteins in 64% of cases, and with lipids in 43% of cases. Almost 60% of all MB binding events occurred with the spike proteins of the viral envelope, whereas the dye practically did not bind to the M proteins, which are present in more than a thousand copies in the coronavirus envelope (Figure 2c,d, Table 1). The interaction of MB with the M proteins is mediated by the presence of negatively neighboring charged lipids (CDL2—1.77%, POPI—1.08%, POPS—0.36%).

On the contrary, MB binds well to the E proteins; however, since they are present in only two copies in the envelope, the percentage of binding relative to the total number of events is small. Note that the E proteins are surrounded by negatively charged lipids, which increases their ability to bind MB. In particular, 7.5% of the MB binding events with the E proteins refer to simultaneous interaction with POPI, and 6.5%—with CDL2. 

As follows from Table 1, a significant portion of MB binds only to the lipid fraction of the envelope. The observed MB binding to neutral lipids palmitoyl oleoyl phosphatidylethanolamine (POPE), palmitoyl oleoyl phosphatidylcholine (POPC), and cholesterol (CHOL) occurs due to their co-localization with negative lipids (POPS, POPI, and CDL2). To calculate the portion of MB molecules that interact with neutral lipids only, but do not interact with negatively charged lipids, we calculated the number of cases where the only partner of MB is one of the neutral lipids. In particular, MB interacts exclusively with neutral lipids in only 3.5% of cases, which is 15 times less than when taking into account the events of interaction of MB simultaneously with negatively charged structural components of the coronavirus membrane. Note that among the negatively charged lipids, the highest percentage of binding to MB was accounted for by CDL2 (28.2%), which has two polar heads and thus becomes an attractive target for MB. However, a third of MB contacts with CDL2 occurred due to simultaneous binding to both CDL2 and POPI.

With an electrostatic attraction energy of 3 kT and higher, we were able to obtain only 14 MB complexes with the coronavirus envelope, since the rate of formation of such complexes turned out to be extremely low. Eleven complexes were formed with S proteins and one with M protein, and two MB molecules were only in contact with lipids.

## 3. Discussion

The envelope of coronaviruses is formed by a lipid bilayer with embedded structural proteins. The envelope membrane originates from the membranes of the endoplasmic reticulum Golgi intermediate compartment (ERGIC) of the host cell, which coats the budding virus particles [36]. The endoplasmic reticulum (ER) plays the central role in the biosynthesis of cellular lipids. In the ER and Golgi membranes, the major lipid is phosphatidylcholine, followed by phosphatidylethanolamine [37]. The membranes of both organelles are also enriched in negatively charged lipids. In particular, phosphatidylinositol represents 11–12% of the total lipid content [37]. In the SARS-CoV-2 membrane model used here, the lipid content was as follows: POPC (59%), POPE (20%), and POPI (10%). The lipids which represented less than 5% were CHOL (4.5%), CDL2 (4.5%), and POPS (2%). In SARS-CoV-2, three of the four major structural proteins—M, S, and E—are parts of the viral envelope [38]. According to [39], the number of the M protein homodimers in the coronavirus envelope was estimated to be 1100 copies. The “corona” appearance of the SARS-CoV-2 virion is formed by 26 ± 15 spikes [40]—the homotrimeric S proteins of about 1300 amino acids and 20 nm in length. The E protein which is able to form the homopentameric viroporin is abundant in the ER, Golgi, and ERGIC membranes of the infected cells [41], but is only presented in a few copies in the virus envelope. The CG model of the SARS-CoV-2 envelope used in our study included all three of these protein types: 1003 copies of the M protein dimers, 25 copies of the glycosylated S protein spikes, and 2 copies of the E protein channels. Thus, the lipid and protein content in the CG model of the SARS-CoV-2 envelope generally reflects their known physiological levels.

Among the structures of coronaviruses, spikes have received increasing attention. These homotrimeric surface glycoproteins play a crucial role in cell receptor recognition, virus binding, and entry into host cells. The spike protein was the first structural protein of SARS-CoV-2 to be proposed as a druggable target [42], and many studies to develop vaccines and therapeutic and diagnostic tools have focused on this protein [41]. 

During the entry of SARS-CoV-2, the first step involves the recognition of the receptor on the host cell membrane and binding to it using the S1 subunit of the S protein, and the second step involves fusion of the viral envelope membrane and the host cell membrane mediated by the S2 subunit of the S protein. In in vitro experiments, when added to viral particles prior to the infection of host cells, MB showed anti-SARS-CoV-2 [13,30] and anti-BCoV [12] activity at very low micromolar concentrations. It was also able to inhibit the entry of a SARS-CoV-2 spike bearing pseudovirus into angiotensin-converting enzyme type 2 (ACE2)-expressing cells and was active in blocking the protein–protein interaction (PPI) between the RBD of the S protein and ACE2 of the host cell [35]. It has been proposed that the inhibition of RBD-ACE2 PPI reduces the efficiency of cell infection, and this mechanism underlies the antiviral activity of MB. The simulation of MB interactions with spikes of three coronaviruses allowed us to reveal a binding site of this dye at the junction of the S protein stalk and head adjacent to the heptad repeat 2 (HR2) domain. This site was common to Severe Acute Respiratory Syndrome-related Coronavirus (SARS-CoV), Middle East Respiratory Syndrome-related Coronavirus (MERS-CoV), and SARS-CoV-2 S proteins [33]. In the SARS-CoV-2 S protein, MB was also found to penetrate into the pocket formed by the RBD in the “open” state, N-terminal domain (NTD), and heptad repeat 1 (HR1) domain. In the present study, we confirmed that more than half of the contacts of MB with the SARS-CoV-2 envelope occur through spike proteins. Based on our simulation experiments [33], we came to the conclusion that the binding of MB to the RBD may affect the interaction of the S protein with the ACE2 receptors on the host cell, thus reducing viral infectivity. This corresponds to the conclusions of the paper [35].

MB also has the potential to affect the fusion process due to the multiple binding sites of MB between (according to amino acid sequence) the RBD and the fusion protein (FP) and just after the FP, as well as adjacent to the HR2 S protein domain [33]. In terms of the influence of MB on the stage of membrane fusion, the ability of this cationic dye to bind to negatively charged lipids appears to be even more important. In our simulations (Table 1), MB readily binds to negatively charged lipids or interacts simultaneously with negatively charged and neutral lipids. Although the energy of the electrostatic complexes obtained in the model (2 kT) is of the same order as the energy of thermal fluctuations, such electrostatic interactions lead to the redistribution of MB molecules and generate the areas with a higher probability of contacts with MB. Despite the fact that we only simulated the interactions of MB with the membrane lipids of the coronavirus envelope, the results obtained, with certain assumptions, can be extended to the interaction of the dye with the membranes of host cells. The driving force for fusion is thought to be a combination of hydrophobic and electrostatic effects [43]. The fusion domain of the SARS-CoV-2 S2 subunit, which is responsible for binding and interacting with lipids in the host membrane, has been shown to interact specifically with electronegatively charged phospholipids [43]. Since the same types of lipids provide binding sites for MB, it is conceivable that MB could potentially have an inhibitory effect on the fusion process by inhibiting the binding of the S2 fusion domain to negatively charged host membrane lipids.

In our version of the computational experiment, each MB molecule was randomly placed inside the volume unoccupied by the virion, after which, it came in motion until the formation of the encounter complex. This corresponds to experimental conditions when the dye is added to the viruses before or immediately after the host cell infection step. However, MB exhibits antiviral effects even when added post infection. In a previous study [30], anti-SARS-CoV-2 MB activity was detected when the dye was added at a time (3.5 h after infection) when the internalization of viral particles into cells was completed, but new viral particles had not yet been formed, that is, it involved the inhibition of stages of the viral life cycle that occur in the host cell. In this regard, we would like to draw attention to the E protein and its pentameric ion channels, viroporins, which are considered as a pharmacological target in the development of antivirals against coronaviruses [41]. Located predominantly in the Golgi, ER, and ERGIC membranes of infected cells, the E protein is involved in the morphogenesis and assembly of the virus and maintains the structure of viral particles through interaction with the M protein [41]. Despite the fact that viral envelopes are formed from the membranes of cellular compartments where the E protein is predominantly expressed, the viral envelopes themselves contain only a few copies of the E protein. However, we found a prominent ring area of negative potential around the entrance in such a viroporin channel which is formed by the exposed E protein glutamate residues and co-localized negatively charged lipids and is highly attractive to MB molecules (Figure 3a). The conductance of the E protein channel depends on the total charge of the host membrane lipids [44] and may be sensitive to changes in charge properties as a result of the binding of cationic MB molecules.

The photodynamic antiviral activity of MB, when the dye is activated by irradiation with appropriate wavelengths in the presence of molecular oxygen, has been known for many years [22,45]. In the photodynamic reactions, photoexcited MB molecules generate reactive oxygen species (ROS) that induce the oxidation of the key biomolecules, primarily proteins and unsaturated fatty acids of viral envelope lipids, as well as viral genetic material. Coronaviruses are enveloped viruses and appear to be very sensitive to photodynamic inactivation [46]. The photodynamic activity of MB has been approved for several coronavirus species [12,30,47]. When BCoV coronavirus particles were incubated with 1 μM of MB for 10 min and irradiated with a 663 nm LED at a dose of 4 J/cm^2^, we clearly observed destructive effects on the virus envelope in electron micrographs [12]. The numerous MB binding sites found on the model SARS-CoV-2 envelope (Figure 2) help to clarify the oxidative destruction of this structure. Indeed, one can see the presence of areas of negative potential sufficient for the binding of the cationic dye molecules both on the spikes and on the membrane (Figure 1 and Figure 2). These localizations of MB molecules help photogenerated ROS to reach their sensitive molecular targets. 

## 4. Materials and Methods

The interaction between MB and the envelope of the SARS-CoV-2 virus was investigated by the BD approach using CG molecular models in the MARTINI force field [48]. The model of the SARS-CoV-2 envelope was adopted from [49] and successively tested in our previous study [34]. The envelope was composed by neutral (11,817 POPE, 34,860 POPC, and 2658 CHOL) and negatively charged (5908 POPI, 1181 POPS, and 2658 CDL2) lipids and embedded proteins (1003 M proteins, 25 S proteins, and two E proteins). The envelope of the SARS-CoV-2 virus exhibits a net positive charge of 8856 elementary charges, primarily attributed to the presence of over a thousand M protein molecules, each carrying a charge of +22 [34]. The E protein is also a positively charged component (+10), whereas, unlike other proteins, the glycosylated S protein carries a negative charge of –33. The negatively charged lipids POPI and POPS have one elementary charge, while CDL2 has two elementary charges. 

A CG molecular dynamics (MD) model of MB was developed on the basis of an all-atom MD model designed using the ATB service [50]. The all-atom structure of MB was mapped into CG particles according to [51]. The CG model of MB consisted of eight particles with a total charge of +1. The bonded parameters of the CG model between interacting particles were refined on the basis of auxiliary all-atom MD simulation by Swarm-CG [52]. To be precise, this software performs a series of short CG MD calculations, subsequently changing the force constants and equilibrium values of angles and bonds until they match the distributions of the same parameters in the all-atom MD calculation. This auxiliary all-atom MD calculation of a single MB molecule in a water box (employing the SPC water model) was performed at a concentration of 150 mM Na^+^/Cl^−^ ions, using the GROMOS96 54a7 force field with a time step of 2 fs, in the NPT ensemble (the V-rescale thermostat at 303.15 K along with a Parrinello–Rahman barostat at 1 bar) during 400 ns [53].

We consider the interaction of the mobile MB molecule with the rigid immobile viral envelope in solution as a multistage process involving free diffusion of the MB molecule in solution, its random approach and spatial orientation, and further approach of oppositely charged regions due to long-range electrostatic interactions. These processes, leading to the formation of an energetically favorable interaction complex with the viral envelope, were simulated using the BD method implemented in the previously developed ProKSim program [54,55]. In the BD method, each mobile molecule is considered as a Brownian particle that performs translational and rotational motions in a viscous medium under the influence of a random force arising from its collisions with the medium and electrostatic forces. To mathematically describe the process of Brownian motion, the Langevin equation is used, which determines the change in each coordinate over time under the influence of random and external forces.

The Poisson–Boltzmann equation was employed to calculate the electrostatic potential field for both the SARS-CoV-2 virion and the MB molecule. The SARS-CoV-2 virion envelope was represented as a low dielectric area (ε = 2) with fixed partial charges. The aqueous solvent (dielectric constant ε = 80) containing ions was treated implicitly with an ionic strength of 100 mM. An electrostatic cut-off radius of 3.5 nm was used.

In the BD simulations, the SARS-CoV-2 virion was placed at the center of a virtual reaction volume with periodic boundary conditions, measuring 200 × 200 × 200 nm. In each simulation run, the MB molecule was randomly positioned within the volume not occupied by the virion. While the virion remained immobile, the MB molecule underwent motion due to both random and electrostatic forces. The simulations continued until the electrostatic attraction energy between the MB molecule and the virion reached a predetermined threshold of 2 or 3 kT. The final position of MB relative to the virion was recorded for subsequent analysis, and the simulation was then restarted with MB placed randomly once again. In total, we generated 33,000 configurations of encounter complexes involving MB and the SARS-CoV-2 virion at the 2 kT threshold. The rate of formation of complexes with an energy of 3 kT was so low that we were able to obtain only 14 structures of such complexes.

To assess interactions between the MB molecule and specific components of the virion, we considered contact to have occurred if any CG bead of MB approached any CG bead of the virion within a distance of 0.6 nm. This distance was chosen on the basis that it should be minimal, but for the vast majority of detected electrostatic complexes, the MB molecule would be in contact with at least one of the components of the coronavirus envelope. With a threshold distance equal to 0.6 nm and electrostatic attraction energy greater than 2 kT, this requirement was met in 99.96% of cases. To characterize the interaction between MB and virion components, we developed a custom Python script using the MDAnalysis library [56]. This script identified and counted the specific components of the viral envelope that came into contact with the MB molecule. Molecular structures were visualized using PyMOL software version 2.5 [57].

## 5. Conclusions

The current state of computational technology allows for the large-scale modeling of interactions of drugs with whole-virion structures, which brings us closer to understanding the mechanisms of drug action and resistance development and allows us to predict new structures of antivirals and identify druggable structures. The binding sites of MB with the coronavirus envelope identified through molecular modeling make both the phototoxicity of this dye and the MB-induced inhibition of coronaviruses in the absence of light clear.

## Figures and Tables

**Table 1:** Contacts of MB molecules with components of the SARS-CoV-2 envelope with energy of electrostatic attraction of more than 2 kT.

Components of the Viral Envelope			Fraction, %
Proteins		S	59.5
		M	3.2
		E	2.0
Lipids	Negatively charged	POPI	17.7
		CDL2	28.2
		POPS	4.3
	Uncharged	POPC	31.0
		POPE	15.2
		CHOL	0.3

## Data Availability

Not applicable.
